# Multi-Locus Sequence Typing of *Bartonella bacilliformis* DNA Performed Directly from Blood of Patients with Oroya’s Fever During a Peruvian Outbreak

**DOI:** 10.1371/journal.pntd.0004391

**Published:** 2016-01-29

**Authors:** Maria J. Pons, Wilmer Silva-Caso, Juana del Valle-Mendoza, Joaquim Ruiz

**Affiliations:** 1 Centro de Investigación de la Facultad de Ciencias de la Salud, Universidad Peruana de Ciencias Aplicadas (UPC), Lima, Peru; 2 ISGlobal, Barcelona Ctr. Int. Health Res. (CRESIB), Hospital Clínic – Universitat de Barcelona, Spain; U.S. Naval Medical Research Unit No. 2, INDONESIA

## Abstract

**Background:**

*Bartonella bacilliformis* is the etiological agent of Carrion’s disease, a neglected tropical poverty-linked illness. This infection is endemic of Andean regions and it is estimated that approximately 1.7 million of South Americans are at risk. This bacterium is a fastidious slow growing microorganism, which is difficult and cumbersome to isolate from clinical sources, thereby hindering the availability of phylogenetic relationship of clinical samples. The aim of this study was to perform Multi Locus Sequence Typing of *B*. *bacilliformis* directly in blood from patients diagnosed with Oroya fever during an outbreak in Northern Peru.

**Methodology/Principal Findings:**

DNA extracted among blood samples from patients diagnosed with Oroya’s fever were analyzed with MLST, with the amplification of 7 genetic loci (*ftsZ*, *flaA*, *ribC*, *rnpB*, *rpoB*, *bvrR* and *groEL*) and a phylogenetic analysis of the different Sequence Types (ST) was performed. A total of 4 different ST were identified. The most frequently found was ST1 present in 66% of samples. Additionally, two samples presented a new allelic profile, belonging to new STs (ST 9 and ST 10), which were closely related to ST1.

**Conclusions/Significance:**

The present data demonstrate that *B*. *bacilliformis* MLST studies may be possible directly from blood samples, being a promising approach for epidemiological studies. During the outbreak the STs of *B*. *bacilliformis* were found to be heterogeneous, albeit closely related, probably reflecting the evolution from a common ancestor colonizing the area. Additional studies including new samples and areas are needed, in order to obtain better knowledge of phylogenetic scenario *B*. *bacilliformis*.

## Author Summary

The bacteria *Bartonella bacilliformis* is the etiological agent of Carrion’s disease, which is a neglected poverty-related disease, related to Mountain Andean valleys of Peru, Colombia and Ecuador. This disease, in absence of treatment presents a high mortality during the acute phase, called Oroya’s Fever. The second phase is characterized by the development of dermal eruptions, known as “Verruga peruana” (Peruvian wart). This bacterium is a fastidious slow growing microorganism, being difficult and cumbersome to isolate from clinical sources. Then, the available data about phylogenetic relationship in clinical samples are really scarce, but suggesting high variability. The aim of the study was to perform direct blood analysis of *B*. *bacilliformis* Multi Locus Sequence Typing (MLST), a genotyping tool, in patients with Oroya fever during an outbreak. The present study demonstrates that the direct blood PCR, followed by nucleotide sequencing and MLST is a technique useful in the phylogenic characterization of this fastidious microorganism endemic from Andean regions. In this study, we demonstrate that the outbreak of Oroya’s fever was caused by closely related Sequence Typing (ST) microorganisms and, additionally, new STs have been described.

## Introduction

Carrion’s disease is neglected tropical neglected poverty-linked illness caused by *Bartonella bacilliformis*. This infection is endemic in low-income areas of Peru, specifically related to Andean regions from Peru, Ecuador and Colombia, covering roughly 145,000Km^2^ only in Peru, and it is estimated that approximately 1.7 million of South Americans are at risk [1–3]. This illness has two phases: the first, named Oroya’s Fever, mainly affects young children (>60% of cases) and is characterized by fever, acute bacteremia at about 60 days and severe hemolytic anemia [2,4]. Complications are common in this phase, and secondary infections are also frequent due to transient immunosuppression [5]. In the absence of adequate treatment, high levels of mortality (44% to 88%) have been reported [2,4]. The second phase is called “Verruga Peruana” (Peruvian Wart), in which the bacterium induces the proliferation of endothelial cells, resulting in a series of cutaneous lesions [6]. A variety of verrugal lesions are presented in the chronic phase: miliary, nodular and mular [1]. Asymptomatic carriers have also been described in the population from endemic areas (0.5–45%) [7].

*B*. *bacilliformis* is a fastidious slow growing microorganism, which is difficult and cumbersome to culture and isolate from clinical sources [2]. Thus, the data available about the phylogenetic relationship of clinical samples of *B*. *bacilliformis* are scarce and non-uniform. Indeed, to the best of our knowledge no studies on clonal relations based on Pulsed Field Gel Electrophoresis (PFGE) have been performed, and molecular approaches have been based on PCR methodologies, including Repetitive Extragenic Palindromic PCR (REP-PCR), Enterobacterial Repetitive Intergenic Consensus (ERIC-PCR), Amplified Fragment Length Polymorphism (AFLP), Infrequent Restriction Endonuclease Site PCR (IRS-PCR), analysis of the 16S-23S ribosomal DNA intergenic spacer regions or analysis of the sequence of specific genetic loci such as *gltA*, *ialB* and *flaA* [8–10]. This latter methodology resembles a Multi-locus sequence typing (MLST) technology. MLST approaches are based on housekeeping gene sequencing, being robust, standardized methodology useful to develop epidemiological and evolutionary studies [11]. In fact, MLST schedules have been developed to analyze the phylogenetic relationships of *Bartonella henselae* [12], and adapted to other *Bartonella* species, including *Bartonella quintana* [13] and *Bartonella bovis* [14]. Furthermore, the use of MLST has been useful in the identification of *Bartonella ancashi*, new specie of *Bartonella* genus, closely related to *B*. *bacilliformis* [15]. Regarding *B*. *bacilliformis*, a MLST schedule has recently been developed based on the sequence of 7 housekeeping genes (*bvrR*, *ribC*, *ftsZ*, *groEL*, *flaA*, *rnP* and *rpoB*) [16], with 8 different ST being detected in 43 isolates. However, it should be considered that due to the relative isolation of the Andean valleys, the population structure of *B*. *bacilliformis* might differ between different endemic areas.

The aim of the study was to perform direct blood MLST of *B*. *bacilliformis* from patients diagnosed with Oroya Fever during an outbreak in Northern Peru.

## Materials and Methods

### Samples

**Fig 1:**
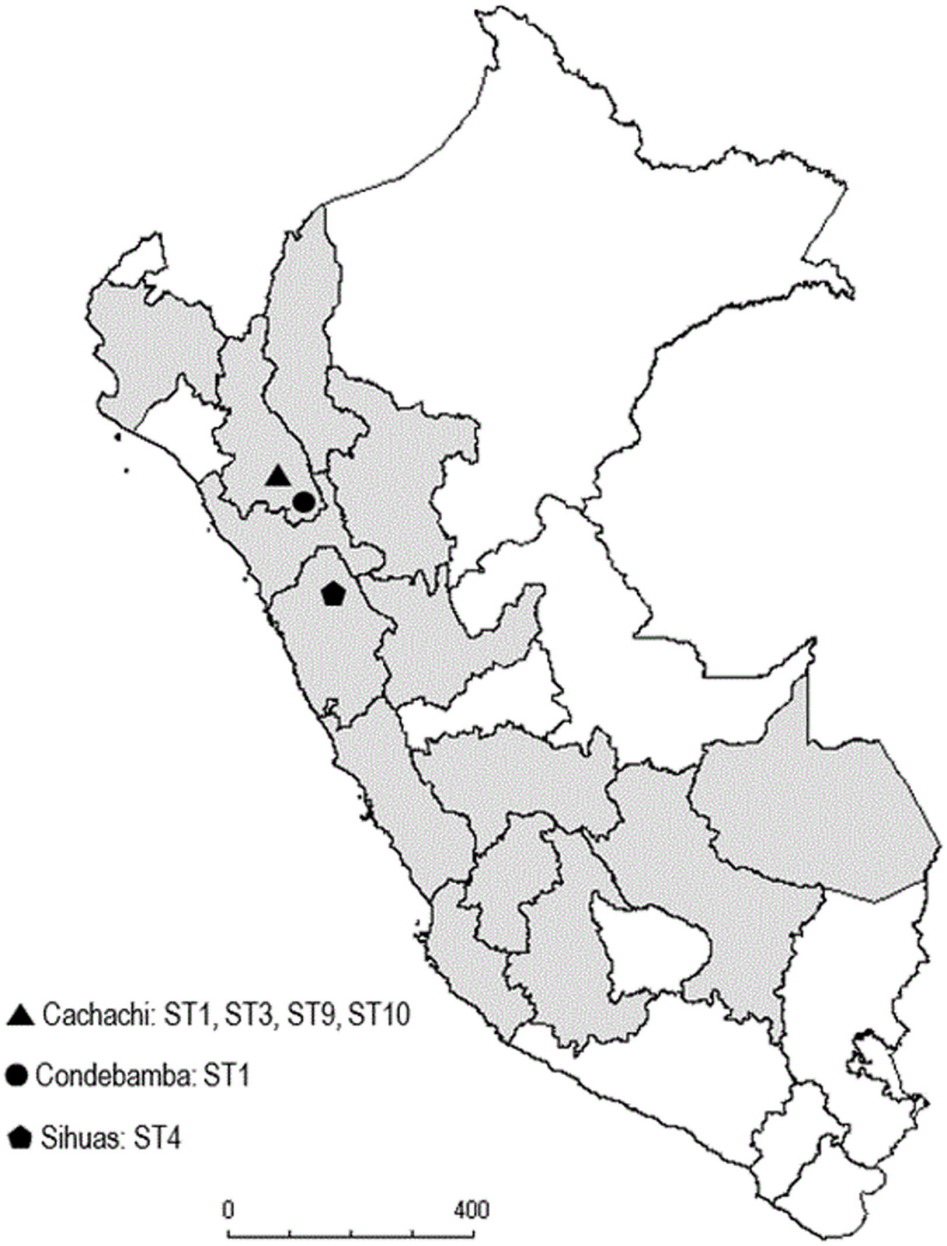
Map of the geographical distribution of Carrion’s disease in Peru with the distribution of the SequenceTypes location.

Seven blood samples from Cachachi (Department of Cajamarca in Northern Peru) were collected during March and April 2009 from patients clinically diagnosed with Oroya Fever. Additionally, another two blood samples were collected from Oroya’s Fever patients living in the Condebamba (Cajamarca Department, 50 Km from Cachachi) and Ancash Department in November and October 2011, respectively. Finally, two collection strains isolated in 1941 (CIP 57.19; NCTC12135) and 1949 (CIP 57.18; NCTC12134) from the Pasteur Institute Collection and previously described as belonging to Sequence Type 3 [16] were used as controls (Fig 1). The clinical data and disease presentation of some patients were obtained.

### Ethical statement

All adult participants provided written informed consent. The study were submitted, revised and approved by the Ethics and Research Committees of the Universidad Peruana de Ciencias Aplicadas in Peru and Hospital Clinic of Barcelona in Spain.

### Detection of *Bartonella bacilliformis*

The presence of *B*. *bacilliformis* in all the blood samples was confirmed by PCR amplification of 438 bp of the *16S rRNA* gene of *B*. *bacillifomis* (5’CCTTCA GTTMGGCTGGATC-3’ and 5’-GCCYCCTTGCGGTTAGCACA-3’) as previously described [17]. In all cases the identity of the amplified fragments was confirmed after being visualized in 1.5% agarose gel stained with Sybr Safe and gel recovered using Wizard SV gel and PCR clean up system, (Promega, Madison, WI, USA) following manufacturer's instructions and were sequenced by Macrogen (Seoul, Korea).

### DNA extraction

The DNA was extracted from 200 μl of blood sample and directly from the control bacterial strains using a commercial extraction kit (High Pure Kit Preparation template, Roche Applied Science, Mannheim, Germany). Blood and bacterial DNA obtained after extraction were eluted in 100 μl of nuclease free water and then processed or stored at -20°C until use.

### MLST genes amplification

Internal fragments of the 7 genetic loci (*ftsZ*, *flaA*, *ribC*, *rnpB*, *rpoB*, *bvrR* and *groEL*) included in the *B*. *bacilliformis* MLST schedule were amplified as previously described [16]. Reaction mixtures were exposed to denaturation at 96°C for 5 min followed by 50 cycles of 96°C for 40 sec, 55°C for 40 sec and 72°C for 50 sec, with a final extension step of 72°C for 10 minutes. Amplified fragments were visualized in 1.5% agarose gel stained with Sybr Safe and subsequently gel recovered using Wizard SV gel and PCR clean up system, (Promega, Madison, WI, USA) following manufacturer’s instructions and sequenced by Macrogen (Seoul, Korea).

### Phylogenetic analysis

Phylogenetic relationship analyses were conducted using MEGA version 5 [18]. The phylogenetic tree was constructed by UPGMA (Unweighted Pair Group Method with Arithmetic Mean Analysis). The phylogenetic tree was inferred from 500 bootstrap replicates. The sequences of all the alleles described previously were obtained from Genbank (accession numbers JF326267 to JF326294) and were ordered according to the corresponding Sequence Type (ST) in order to develop the phylogenetic tree.

## Results

The mean age of patients studied was 25.9 years (SD = 13.77, IC_95%_ = 19.5–32.3), 44.4% being female. Among the 5 patients from whom clinical data were recovered, all (100%) presented fever (>38°C) and malaise, 4 (80%) reported chills, myalgia and pallor, 3 (60%) headache, 2 (40%) reported jaundice and arthralgia and only one patient (20%) presented vomiting (Table 1). In 3 cases the treatment was recorded, in all cases being ciprofloxacin alone (2 cases) or with ceftriaxone (1 case) during 14 days.

**Table 1:** Clinical and epidemiological characteristics of Oroya Fever infected patients collected during the outbreak in Northern of Peru.

Sample ID	Carrion phase	Age	Gender	Fever	Malaise	Chills	Myalgia	Arthralgia	Headache	Vomiting	Pallor	Jaundice
EC 1	Acute	15 years	Male	ND	ND	ND	ND	ND	ND	ND	ND	ND
EC 2	Acute	25 years	Female	+	+	+	+	+	+	+	+	-
EC 8	Acute	16 years	Female	ND	ND	ND	ND	ND	ND	ND	ND	ND
EC 14	Acute	33 years	Male	+	+	+	+	-	+	-	+	-
EC 35	Acute	47 years	Male	+	+	+	+	+	+	-	-	-
EC 44	Acute	32 years	Male	ND	ND	ND	ND	ND	ND	ND	ND	ND
EC 48	Acute	35 years	Female	ND	ND	ND	ND	ND	ND	ND	ND	ND
EC 125	Acute	29 days	Male	+	+	-	-	-	-	-	+	+
EC 129	Acute	30 years	Female	+	+	+	+	-	-	-	+	+

ID: Identification; ND.- Non determined

Among the 9 blood samples analyzed, a total of 4 different *B*. *bacilliformis* STs were identified. The most frequently found was ST1, present in 6 out of 9 (66%) samples, all from the Cajamarca Department (5 out of 7 belonging to the Cachachi outbreak, and that of Condebamba), while the sample from the Ancash Department belonged to ST4 (Fig 1). Additionally, two samples from the Cachachi outbreak presents a new allelic profile, belonging to new STs, which were classified as ST9 (1,2,1,1,1,1,1) and ST10 (1,1,1,1,1,3,1) respectively. The 2 collection strains were classified as ST3 (Table 2).

**Table 2:** Multi-locus sequence typing (MLST) information of Oroya Fever samples.

			MLST allelic profile							
Sample ID	Locality, District, Department	Date	ftsZ	flaA	ribC	rnpB	rpoB	bvrR	groEL	ST
1	Carrizal, Cachachi, Cajamarca	march-09	1	1	1	1	1	3	1	ST10
2	Chuquibamba, Cachichi, Cajamarca	march-09	1	2	1	1	1	1	1	ST9
8	Shirac, Cachachi, Cajamarca	march-09	1	1	1	1	1	1	1	ST1
14	Shirac, Cachachi, Cajamarca	march-09	1	1	1	1	1	1	1	ST1
35	Pampa Mirador, Cachachi, Cajamarca	march-09	1	1	1	1	1	1	1	ST1
44	Moncada, Cachachi, Cajamarca	march-09	1	1	1	1	1	1	1	ST1
48	Picachos, Cachachi, Cajamarca	may-09	1	1	1	1	1	1	1	ST1
125	Pisgullo, Sihuas, Ancash	oct-11	1	2	2	3	1	3	3	ST4
129	Huañinba, Condebamba, Cajamarca	nov-11	1	1	1	1	1	1	1	ST1
c57.17	Collection strain	-	1	2	2	3	1	2	3	ST3
c57.18	Collection strain	-	1	2	2	3	1	2	3	ST3

ID: Identification; ST: Sequence Typing

**Fig 2:**
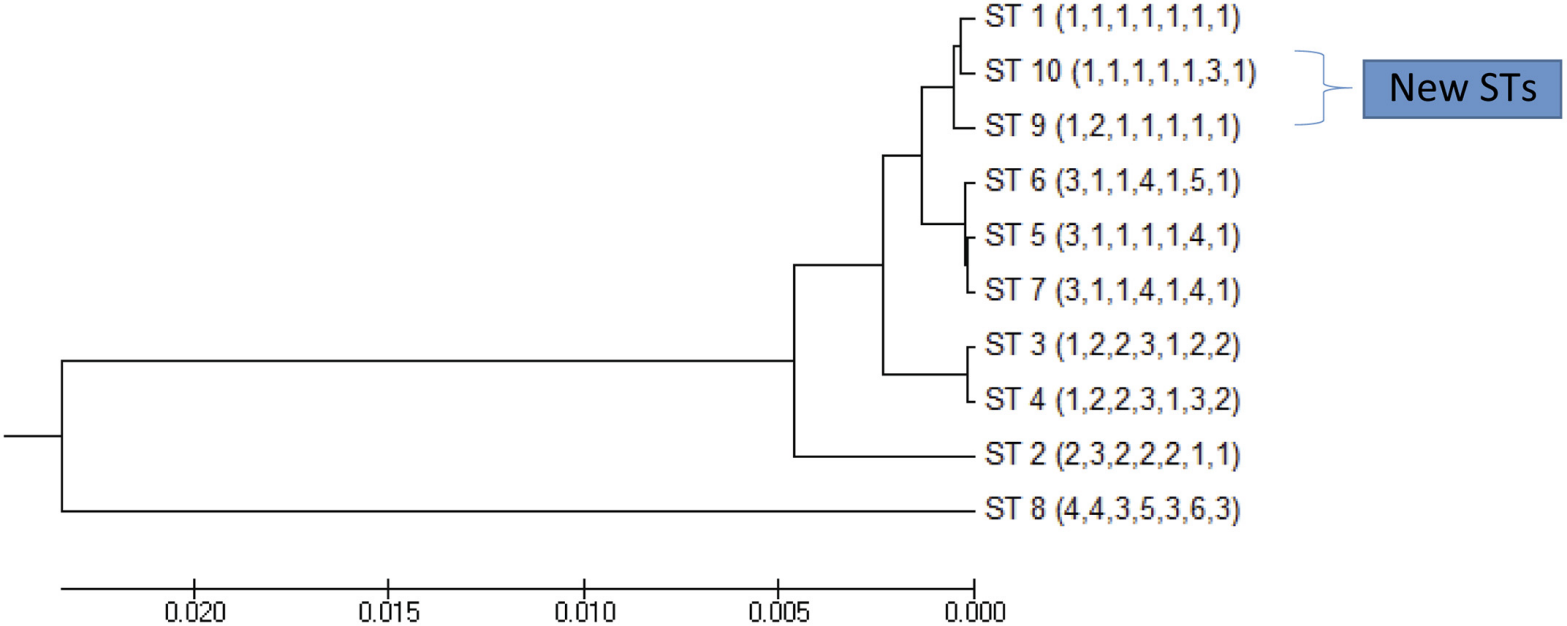
Phylogenetic tree of all the Sequence Types of *B*. *bacilliformis* as constructed an UPGMA cluster analysis with Bootstrap method.

On determination of phylogenetic relationships between the ST9 and ST10 and the previously described ST, they were found to be closely related to ST1, differing in only 1 of the 7 alleles (Fig 2).

## Discussion

Studies on the clonality and phylogeny of *B*. *bacilliformis* are scarce. This may be due to the slow growth of this bacterium and a series of specific requirements which directly affect the culture. The present study demonstrates MLST studies of *B*. *bacilliformis* may be performed directly from blood samples thereby avoiding the difficult step of culturing this microorganism. However, a series of limitations that may limit the usefulness of this technique should be taken into account. Among these, definitive results may not be obtained in the hypothetical case of polyclonal infections when the infecting isolates belong to different STs. Along this line, although to the best of our knowledge coinfection by two different *B*. *bacilliformis* clones has not been described to date, coinfections by different *Bartonella* variants has been reported in cotton rats [19]. In the present report, new MLST were not related to artefactual overlapping of sequences belonging to different *B*. *bacilliformis* causing a concomitant infection, because no double peaks were observed in any DNA sequence. Another limitation is that direct blood PCR approaches in the study of asymptomatic *B*. *bacilliformis* carriers do not have enough power due to the low bacterial burden [20].

The present study demonstrates the heterogeneity of the *B*. *bacilliformis* population. Highly clonality has also been found in other species of *Bartonella*, such as *B*. *quintana*, a re-emerging pathogen causing trench fever [13]. Thus, 3 different ST (ST1, ST9 and ST10) were recovered amongst the samples analyzed from the Cachachi outbreak. However, it is of note that these 3 STs were closely related to each another, and thus may reflect the evolution from a common ancestor colonizing the area.

To date only one study has determined the MLST of *B*. *bacilliformis* isolates [16]. In this study ST1 was found to be widely distributed in central and northern areas of Peru, accounting for 46% of the samples analyzed, including samples from the 1960's. In addition, ST1 has been detected in the neighboring San Martin Department, and thus, its presence in the Cajamarca Department is not surprising. ST2, ST3, ST4 and ST8 have been described in the center of the country, similar the present sample belonging to ST4. Meanwhile, ST5 has been observed in southern isolates and ST6 and ST7 in the north of the country.

Some techniques such as PFGE or REP-PCR are useful for the description of clones and specific outbreak characterization, as for example virulence, being of special interest to study recent genetic events. On the other hand, techniques such as MLST classification describe ancient genetic differentiations that may underlie more in depth differences [11–13]. For example, specific STs could possess increased virulence or may have a greater facility to develop either acute or chronic infection, or to remain undetected in asymptomatic carriers. Unfortunately, the scarce data on STs of *B*. *bacilliformis* make it difficult to delineate these aspects.

The clinical data of only a few patients were recorded; however the symptoms reported are in accordance with those more extensively described, such as the presence of fever, pallor, malaise and headache in acute cases, [17]. Some of these symptoms are a consequence of hematological complications, such as pallor, while others like vomiting or jaundice are related to gastrointestinal problems [21]. Although this disease mainly affects children under 14 years of age (more than 60% of cases) [22], in our study the youngest patient of the Cachachi outbreak was 15 years old. This may be related to the outbreak nature of the samples. Fortunately, all patients who receipt treatment respond well to the treatment. The currently recommended treatment for the acute phase of Oroya’s Fever includes the use of ciprofloxacin as first line therapy in adults and children >14 years, while chloramphenicol, cotrimoxazole, amoxicillin plus clavulanic acid and ceftriaxone are used as a second line or children and pregnant women [23]. Fortunately, up to now, the levels of antibiotic resistance reported among *B*. *bacilliformis* have shown that this microorganism is highly susceptible to the antibiotics tested [24].

In summary, this is the first report of MLST of *B*. *bacilliformis* performed in direct blood samples, with two new ST variants being described. Present data highlight the need to extend the studies to new samples and geographical areas, in order to provide a better picture of the situation, which will allow specific STs of *B*. *bacilliformis* to be associated with clinical symptoms, and the severity or phase of the disease.
